# Rapamycin as longevity enhancer and cancer preventative agent in the context of p53 deficiency

**DOI:** 10.18632/aging.100494

**Published:** 2012-10-27

**Authors:** Lawrence A. Donehower

**Affiliations:** Departments of Molecular Virology & Microbiology, Molecular & Cellular Biology, and Pediatrics, Baylor College of Medicine, Houston, TX 77030

Since initial rodent studies in the mid-1930s, caloric restriction (CR) has been known to be an effective non-pharmacological intervention that can extend longevity in many species. Over the last 15–20 years, studies in yeast, worms, and flies have defined many of the signaling pathways mediating these CR-driven longevity effects. A prominent mediator of CR is the target of rapamycin (TOR) signaling pathway, which functions to monitor nutrient levels in the cell and modulate protein synthesis and cell growth in response. Dysfunctional regulation of TOR in humans has been associated with a number of aging-associated diseases such as diabetes, obesity, cardiovascular disease, and cancer [1]. On the other hand, downregulation of the TOR pathway in yeast, worms and flies by the inhibitory molecule rapamycin has been shown to significantly increase lifespan in each of those species. Moreover, in 2009, Miller, Harrison and colleagues showed that mixed inbred mice treated with rapamycin at a relatively late age (600 days) exhibited extended lifespans [2]. Recently, this group also showed that aging phenotypes were significantly delayed in the rapamycin-treated group, though testicular degeneration and cataracts increased [3].

Despite potential side effects, the rapamycin-induced longevity enhancement in a mammalian species has generated much excitement, and further studies in animal models have now indicated that cancer incidence is delayed by rapamycin treatment. This should not be too surprising, since rapamycin integrates signals initiated from a number of growth factor receptors, is upregulated in numerous cancers, and has been used as a cancer therapeutic drug in some contexts [1]. One such study by Anisomov et al. [4] showed that rapamycin treatment of HER-2 transgenic mammary cancer prone mice not only resulted in significantly extended lifespans, but also dramatically delayed tumor appearance and decreased tumor number and size. Thus, rapamycin may be a highly effective cancer preventative drug in addition to its many other beneficial effects.

To follow up and extend this initial exciting result, Gudkov, Blagosklonny, Antoch, and their colleagues have investigated the effects of rapamycin on tumor incidence and longevity in p53-deficient mice. The results, appearing in two papers in this issue of *Aging*, confirm that the cancer preventative effects of rapamycin are significant and broad in scope [5,6]. The p53 tumor suppressor protects against an array of different tumors and p53-deficient mice succumb to lymphomas and many different types of sarcomas [7]. In the paper by Komarova et al. [5], mice heterozygous for a germline p53 null allele (p53+/−) that were continuously treated with rapamycin in the drinking water beginning at a young age (<5 months) had a mean lifespan of 480 days compared to that of the control group's 373 day mean lifespan (a 28% increase). Importantly, these rapamycin-treated mice developed only half as many tumors as the control mice, a dramatic and significant anti-cancer effect. They also show direct inhibition of mTOR kinase activity in several tissues of the rapamycin-treated mice, an indicator that the rapamycin effects continuously inhibit mTOR signaling. In their discussion, the authors acknowledge that the anti-cancer effects of rapamycin are likely to be indirect, but don't speculate further. However, because mTOR integrates signals from so many growth signaling pathways, intersects with so many key growth signal transducers (such as AKT, PI-3 kinase, and Ras), and drives so many cell growth outputs, it's easy to argue that reduction of mTOR activity by rapamycin acts as a major brake on transformation. The authors suggest that the dramatic effects of rapamycin on p53+/− mice could lead to use of this agent as a cancer preventative drug in Li-Fraumeni syndrome patients. Li-Fraumeni patients are analogous to p53+/− mice, as they carry germline p53 mutations and are highly cancer prone at a young age [8]. This may be a good place to start in considering patients for rapamycin in clinical trials, though some of the side effects of rapamycin in mice indicated above [3] certainly need further evaluation.

In the second paper by Comas et al. [6], the authors treat p53 null (p53−/−) mice with rapamycin from the age of 8 weeks. These mice are profoundly tumor prone and succumb to lymphomas by 4–6 months of age. In this paper, however, the bioavailability of the relatively insoluble rapamycin was enhanced by a novel rapamycin formulation called Rapatar that improved water solubility. The authors showed that blood rapamycin levels were significantly increased in animals treated with Rapatar compared to the standard form of rapamycin. As with the p53+/− mice, Rapatar treatment of the p53−/− mice resulted in significant longevity extension and delayed cancer formation relative to untreated p53−/− mice. Mean tumor latencies for the control p53−/− mice and the Rapatar-treated p53−/− were 161 and 261 days, respectively, a very significant effect. The authors argue that improvement of rapamycin bioavailabity through improved formulations is a necessity for clinical applications. They are uncertain whether the rapamycin effects are direct or indirect, but believe it to delay tumorigenesis by slowing aging. However, because the p53−/− mice are relatively young when they develop tumors, this interpretation seems less likely. Nevertheless, both papers represent exciting new advances that could lead us closer to pharmaceuticals that both enhance lifespan and delay cancer.
